# Inflammation and Cardiovascular Diseases: The Most Recent Findings

**DOI:** 10.3390/ijms20163879

**Published:** 2019-08-09

**Authors:** Daniela Sorriento, Guido Iaccarino

**Affiliations:** Department of Advanced Biomedical Sciences, Federico II University, Via Pansini 5, 80131 Napoli, Italy

**Keywords:** inflammation, NFκB, cytokines, inflammatory diseases, heart failure, cardiovascular risk

## Abstract

The series of reactive biological events that we identify as inflammation has been investigated in recent years and unveiled as an important mechanism for regeneration. The study of the underlying complexity has been boosted by new technological innovation in research and allowed the identification of inflammatory responses as the basis of diseases that were considered degenerative rather than regenerative in nature. This is the case for cardiovascular diseases, from the organ damage that follows an acute event to the damage of target organs exposed to chronic risk factors. This editorial explores innovative aspects of inflammation in the setup of cardiovascular risk factors and diseases.

## 1. Introduction

Cardiovascular diseases represent the main cause of death worldwide. To date, studies in this field have allowed the identification of the key molecular mechanisms that regulate cardiovascular function leading to the development of new therapeutic drugs. In this context, recent evidence suggests that immune cells are a key player in the development of cardiovascular diseases and are potential targets for treatments. Indeed, inflammation is the trigger of the early phases of the atherosclerotic process and an increase of inflammatory cytokines is associated with a higher risk of developing cardiovascular diseases. The key role of innate immunity in cardiovascular diseases was uncovered in the Cantos study [1]. This clinical trial demonstrated that targeting of interleukin 1β using a specific monoclonal antibody (Canakinumab) lowers the rate of recurrent cardiovascular events in patients with previous myocardial infarction and high levels of C-reactive protein and these effects are also associated with a decrease of IL-6 levels [1]. The special issue, entitled “Mechanisms of inflammation in degenerative cardiovascular conditions”, presents 14 contributions that highlight the recent advances in the mechanisms of inflammation in cardiovascular diseases. In particular, these studies sustain the proof of concept that inflammation represents a significant cardiovascular risk factor [2,3,4,5] and is also mandatory to the development of both cardiac [2,3,5,6,7,8,9,10,11] and vascular events [4,12,13,14,15], and can be targeted using specific and non-specific therapeutics [8].

## 2. Inflammation as a Cardiovascular Risk Factor

It has been demonstrated that the levels of inflammatory cytokines are increased in patients with heart failure [2]. Moreover, inflammatory markers (i.e., PCR levels) predict a worse survival during acute coronary syndromes [2]. Accordingly, cardiovascular risk increases in patients with chronic inflammatory diseases such as rheumatoid arthritis [2], systemic lupus erythematosus [4], periodontitis [3], and atopic dermatitis [5]. Mercurio and colleagues showed that in patients with lupus erythematosus the increased arterial stiffness significantly correlates with several markers of inflammation [4]. Liccardo suggested that individual with periodontitis had a higher risk of developing cardiovascular events including myocardial infarction, endothelial dysfunction, peripheral artery disease, stroke, and heart failure [3].

Varricchi [5] and colleagues showed that human IgG anti-IgE isolated from the serum of a patient with atopic dermatitis can bind and activate primary mast cells isolated from human myocardial tissue (HHMC) through the release of pro-inflammatory, angiogenic, and lymphangiogenic mediators. Going to the molecular mechanisms of the association between cardiovascular risk and inflammation, the contribution from Fiordelisi and colleagues [2] summarizes the findings that propose the role of the transcription factor NFκB as a bridge between these pathological conditions. On the same note, another paper of this special issue identifies the key role of NFκB: Wang and colleagues [15] showed that miRNA-17, whose expression in VSMC is regulated by NFκB activation, induces VSMC proliferation and is a potential therapeutic target for atherosclerosis, and confirm the key role of NFκB in the crosstalk between inflammation and cardiovascular diseases.

These contributions support the proof of concept that inflammation represents a risk factor for the development of cardiovascular events.

## 3. Inflammation and Vascular Diseases

In this special issue, the mechanisms of inflammation in vascular damage is illustrated by Jin et al. [13], who found that the newly identified suppressor of cytokine signaling, MCP-1-induced protein (MCPIP), is also expressed in endothelial cells and that its deletion is associated with an increased inflammatory phenotype. Thus, this protein seems to be essential to prevent inflammation-dependent endothelial dysfunction and consequently vascular diseases. Besides its role as a trigger of vascular damage, inflammation could also be a consequence of vascular diseases, as it occurs in the brain. Doncow and colleagues [12] suggest that in a mouse model of angiotensin II-induced hypertension, there is an increase of cerebral sphingosine-1-phosphate levels that in turn induces an increase of inflammation as suggested by the elevated CD3+ T-cell number in the brain.

Inflammation is also associated with the progression of abdominal aortic aneurysm (AAA). In patients, low and medium aortic aneurysm calcification indexes are associated with increased inflammatory markers (including CD4, CD20, CD68, MMP-9, IL-6, osteopontin) and oxidative stress-related proteins [14]. This leads to elastic fiber breakdown and the depletion of VSMC, causing aortic wall dilation. In vitro in endothelial cells, the physiological vascular wall stretching exerts an anti-inflammatory effect, which leads to the inhibition of abdominal aortic aneurysm progression [14].

## 4. Inflammation and Cardiac Dysfunction

At the cardiac level, crosstalk between cardiac damage and inflammation is suggested by Pollard [11]. In cardiomyoblasts with osteopontin (OPN) gene deletion, there is a β2AR-dependent upregulation of EPAC-1, an inhibitor of cardiac fibrosis. Thus, the authors propose OPN blockade as a novel therapeutic strategy to counteract cardiac damage, even if further studies are needed to confirm this mechanism in an animal model of cardiac damage.

Cardiac damage secondary to other conditions, such as diabetes, is also in part dependent on the activation of an inflammatory phenotype. Indeed, Cipolletta [7] demonstrated that in db/db mice, which develop diabetic cardiomyopathy, a peptidic inhibitor of the G protein-coupled receptor kinase type 2 (GRK2) corrects the inflammatory phenotype, representing a useful therapeutic target for both diabetes and its cardiovascular complications. This is in agreement with a previous study that demonstrated that the inhibition of GRK2 ameliorates cardiac function in a mouse model of hypertrophy through the inhibition of NFκB signaling [16].

A different role of inflammation in cardiac damage is suggested by La Rocca and colleagues [9]. Cardiac-specific CXCR4 knockout mice are known to develop hypertrophy and cardiac dysfunction in response to chronic catecholamine exposure, but in the absence of exogenous stress, these mice develop progressive cardiomyopathy, leading to heart failure. 

## 5. Therapeutic Targeting of Inflammation in Cardiovascular Diseases

The most common drugs that are used in the clinic for the treatment of cardiovascular disease also exert anti-inflammatory effects, as discussed in the contribution from De Angelis [8]. Beta-blockers, RAAS, and neprilysin inhibitors, despite their effects on cardiomyocytes, all decrease inflammatory cytokine release [8], suggesting that a specific immunomodulatory therapy could be a potential strategy for the treatment of heart failure.

## 6. Conclusions and Future Perspectives

Altogether these findings support the pivotal role of inflammation in the development and progression of both cardiac and vascular diseases, and some of the findings also suggest specific molecules that could be potential targets for therapeutics (NFκB, GRK2, MCPIP, OPN). Several studies have already confirmed that both the direct [17] and indirect [16] inhibition of NFκB ameliorates cardiac function in pathological conditions. However, further studies will be needed to evaluate whether targeting MCPIP and OPN could favor cardiac and vascular functions in pre-clinical models of pathologies. Moreover, several questions should still be elucidated. For example, which type of immune cell could affect cardiac cell function? What are the specific inflammatory molecules that are secreted by these cells, and how do they act within the cell? Future investigations should focus on these issues in order to better understand the crosstalk between inflammatory and cardiac cells in pathological contexts and to identify novel therapeutic strategies. 
